# Age-Dependent Evolution and Synergistic Damping Mechanisms of XSBRL–Rubber-Modified Cementitious Composites

**DOI:** 10.3390/ma19153327

**Published:** 2026-08-05

**Authors:** Jiyang Wang, Shuyu Lin, Qiuyan Jiang, Yu Peng, Jingwen Shi, Junxia Li, Bo Zhang

**Affiliations:** 1 Institute of Advanced Engineering Structures, Zhejiang University, Hangzhou 310058, China; 2 Institute of Materials Research and Engineering, Agency for Science Technology and Research, Singapore 138634, Singapore; 3 Ocean Research Center of Zhoushan, Zhejiang University, Zhoushan, Zhejiang 316021, China

**Keywords:** carboxylated styrene-butadiene rubber latex, chlorinated rubber, modified cementitious composite, damping properties, microstructure, synergistic effect

## Abstract

Incorporating viscoelastic inclusions enhances the damping capacity of cementitious composites, but is often hindered by strength degradation and weak interfacial bonding. This study addresses this trade-off by investigating the synergistic modification of a cement matrix using carboxylated styrene-butadiene rubber latex (XSBRL) and chlorinated rubber (CR) powder, focusing on the age-dependent evolution of their joint energy-dissipation mechanisms. Macroscopic mechanical and microscopic test results reveal a pronounced synergy between latex and rubber powder, governed by possible interfacial interaction. The XSBRL film formed during hydration improves the compatibility between chlorinated rubber and the cement matrix, while its active groups further strengthen the bonding with hydration products. This interfacial coupling transforms the conventionally brittle transition zone into a ductile, high-friction network that maximizes dynamic stress transfer. Moreover, the temporal evolution of damping is governed by the competitive kinetics between cement hydration and polymer-film coalescence, shifting from early-age restructuring (7 to 14 days) to late-stage stabilization (28 days). To balance mechanical and dynamic properties, a recommended formulation of 10% XSBRL and 10% chlorinated rubber is established. This work provides a reference for clarifying the structure–property relationship of high-damping cementitious composites and for optimizing their mix designs.

## 1. Introduction

The escalating vulnerability of modern civil infrastructure, such as high-rise buildings and long-span bridges, to extreme dynamic events has shifted structural design paradigms from traditional stiffness-reliance to active energy-dissipation strategies [1,2]. Portland cement composites, the most ubiquitous construction materials, suffer from low intrinsic damping and high brittleness, rendering them suboptimal for dynamic vibration attenuation under cyclic loading [3]. Developing novel cementitious materials that simultaneously exhibit excellent dynamic energy dissipation and mechanical robustness remains a critical engineering frontier.

To introduce viscoelastic shear hysteresis and micro-interfacial friction into the rigid hydration matrix, incorporating elastomer particles has emerged as an effective modification strategy [4]. Among various polymeric modifiers, rubber powder is highly promising due to its exceptional viscoelasticity, chemical stability, and high density of polar chlorine groups [5,6]. Unlike traditional non-polar rubber aggregates, the polar nature of chlorinated rubber (CR) enhances its thermodynamic compatibility with cement hydration products [7]. Nonetheless, incorporating CR as a discrete powder phase inevitably introduces micro-interfaces and localized microvoids, which disrupt matrix continuity and lead to severe mechanical deficits [8]. This inescapable trade-off between dynamic damping performance and static load-bearing capacity restricts the practical structural applications of CR-modified composites [9].

To mitigate interfacial weaknesses, the introduction of functional polymeric latexes for targeted microstructural repair has gained widespread attention [10,11]. Carboxylated styrene-butadiene rubber latex (XSBRL) is recognized as an effective modifier; its active carboxyl groups coordinate with calcium ions in the pore solution to form stable ionic bridges, while its in situ coalescence forms a continuous viscoelastic film [2,12]. SBR-based latexes have been shown to refine pore structures [13] and dissipate dynamic strain energy through the viscous shear of macromolecular chains [14]. Implementing a secondary modification using XSBRL in CR-modified matrices presents a highly synergistic pathway: the coalesced XSBRL film can physically encapsulate and chemically anchor the CR powder, utilizing their inherent polar–polar compatibility to establish an integrated, multiscale viscoelastic network [15]. Despite the promising performance of such polymer–rubber hybrid systems, their age-dependent evolution and synergistic damping mechanisms remain poorly understood. The dynamic damping of cementitious materials is fundamentally a multiscale phenomenon [16], integrating interfacial viscoelastic shear [17], pore-confined fluid viscous friction [18], and intrinsic matrix friction [19]. During cement hydration, the progressive stiffening of the cement paste competes with the gradual coalescence of XSBRL films and their chemical coupling with CR powder. How these competitive kinetics govern the microstructural topology and interfacial compliance across varying hydration ages has yet to be systematically elucidated.

To address these gaps, this study investigates the mechanical performance, dynamic damping capacity, and pore-structure evolution of cementitious composites modified with XSBRL and CR-10 powder. A two-factor full factorial design is employed to monitor these properties across curing ages of 7, 14, and 28 days. Using mercury intrusion porosimetry (MIP), scanning electron microscopy (SEM), and dynamic mechanical analysis (DMA), we elucidate the age-dependent structural evolution and demonstrate that the synergistic coupling of XSBRL and CR-10 contributes to achieving a more favorable balance between damping enhancement and mechanical performance, establishing a structure–property framework for designing high-performance cementitious composites.

## 2. Materials and Methods

### 2.1. Raw Materials

To systematically investigate the age-dependent evolution and synergistic damping performance of the modified cementitious composites, P·II 52.5 Portland cement (Tongling Conch Cement Co., Ltd., Tongling, China) was used as the primary binder. Precision-graded quartz sand (maximum particle size of 653 μm) was utilized as the fine aggregate. The grain-size distribution of the aggregate was strictly controlled to suppress localized stress concentrations and eliminate microstructural heterogeneity caused by poor gradation, thereby establishing a highly uniform baseline cementitious matrix.

**Figure 1:**
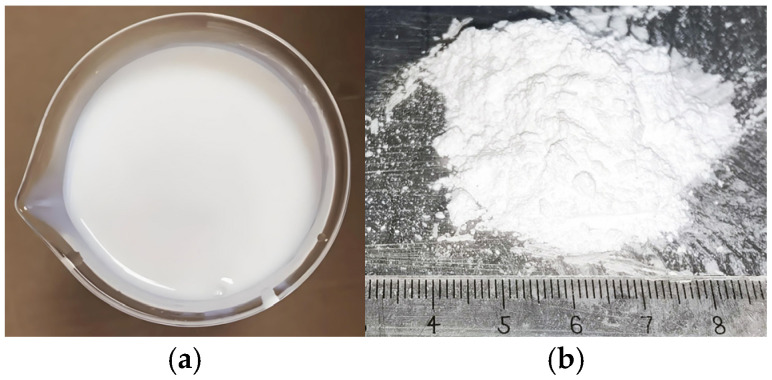
Photographs of the raw polymeric modifiers: (**a**) XSBRL; (**b**) chlorinated rubber powder.

The synergistic damping architecture was constructed by incorporating two distinct functional polymeric modifiers: a carboxylated styrene-butadiene rubber latex (XSBRL) and a chlorinated rubber (CR) powder. The XSBRL (SD623, BASF SE, Ludwigshafen, Germany) was strategically selected for its rapid film-forming kinetics and robust interfacial bridging capabilities within the highly alkaline hydration environment. The CR powder (CR-10, Jinan Quanxin Chemical Co., Ltd., Jinan, China) was introduced as a high-performance polymer filler. Benefiting from its elevated chlorine content (65–68%), the strong polar carbon–chlorine (C–Cl) bonds in CR-10 enhance intermolecular interactions and internal molecular friction under dynamic shear, thereby promising superior viscoelastic energy dissipation when coupled with the elastic network of XSBRL. The macroscopic morphologies of the XSBRL and CR-10 are shown in Figure 1, and their physical and chemical specifications are detailed in Table 1 and Table 2, respectively.

To guarantee sufficient workability at a constant water-to-binder (w/b) ratio, a powder-type polycarboxylate ether-based superplasticizer (PCE, Melflux 2651F, BASF SE, Ludwigshafen, Germany) was employed. Additionally, to counteract the air-entraining tendency inherent to latex polymers and prevent the formation of strength-limiting macropores, a proprietary polyether defoamer (P803, Guangzhou Dachuan Fine Chemical Co., Ltd., Guangzhou, China) was incorporated.

### 2.2. Design of Mixture Proportion

An experimental matrix comprising 14 distinct mixtures was designed to isolate and decouple the individual and synergistic damping contributions of the XSBRL and CR-10 powders. A plain cement mortar—formulated with a water-to-cement (w/c) ratio of 0.30 and a sand-to-cement (s/c) ratio of 2.0—served as the baseline control (designated as S0R0) to characterize the intrinsic behavior of the unmodified cementitious matrix. PCE was maintained at 11.25 kg/m^3^ in all modified mixtures as an auxiliary dispersing admixture. The defoamer was used only in XSBRL-containing mixtures to suppress latex-induced foaming, with its dosage adjusted within a narrow range according to the observed foaming tendency.

The modified mixtures were structured based on a two-factor partial factorial design. The XSBRL was incorporated at mass fractions of 8%, 10%, and 12% by weight of the cement, corresponding to effective active polymer-to-cement (P/C) ratios of 4%, 5%, and 6%, respectively (denoted as S8, S10, and S12 series). To strictly maintain the target effective w/c ratio of 0.30 across all mixtures, the batch water calculation was mathematically compensated. Specifically, the free water introduced via the aqueous phase of the latex (50% wet mass) was quantitatively deducted from the added mixing water. Concurrently, the CR-10 powder was introduced as an equal-mass substitution for the fine aggregate. Aggregate replacement levels were established at 5%, 10%, and 15% by mass of the total aggregate, denoted as R5, R10, and R15, respectively. This replacement strategy was selected because it has been widely adopted in previous studies on rubber-modified cementitious materials and enables direct comparison with published results [9,10].

It should be noted that the primary objective of this study was to investigate the synergistic influence of XSBRL and rubber powder on the damping performance of cementitious materials rather than to establish a statistically complete design of experiments (DOE). Therefore, while all four rubber replacement levels (0%, 5%, 10%, and 15%) were considered under each XSBRL dosage, only the control (0%) and the maximum rubber replacement level (15%) were included for the XSBRL-free mixtures. This experimental arrangement was adopted to reduce redundant testing while maintaining sufficient comparison between the individual and combined modification effects.

This comprehensive matrix established single-doped and hybrid-modified systems, enabling a robust quantitative evaluation of the macro-energy dissipation and corresponding microstructural evolution. The definitive compositional proportions of all 14 mixtures are detailed in Table 3. Alphanumeric codes (e.g., S8R5) uniquely identify the respective XSBRL dosage (S, wt.%) and CR-10 powder replacement level (R, wt.%).

### 2.3. Experimental Methodology

To systematically elucidate the individual and synergistic influences of XSBRL, CR-10 powder, and curing age on the mechanical strength, dynamic viscoelastic damping capacity, and multiscale microstructural evolution of the modified cementitious composites, a comprehensive experimental program was executed.

#### 2.3.1. Static Mechanical Performance Tests

**Figure 2:**
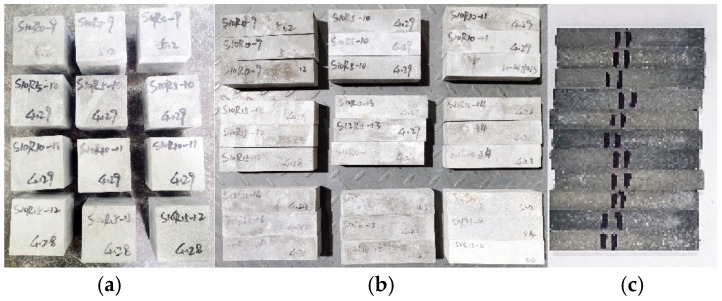
Prepared specimens for mechanical and damping characterization: (**a**) cubic specimens for compressive strength tests; (**b**) prism specimens for flexural strength tests; (**c**) strip specimens for dynamic mechanical analysis (DMA).

To systematically evaluate the macroscopic mechanical performance of the modified cementitious composites, compressive and flexural strength tests were executed using a 1000 kN high-performance fatigue testing machine (Instron 8805, High Wycombe, UK). Compressive strength evaluation was conducted on cubic specimens (70.7 mm × 70.7 mm × 70.7 mm, Figure 2a) in strict accordance with the Chinese standard, Standard for Test Method of Basic Properties of Construction Mortar (JGJ/T 70-2009) [20]. Flexural testing was performed using standard prismatic specimens (40 mm × 40 mm × 160 mm, Figure 2b) in accordance with the Chinese standard Test Method of Cement Mortar Strength (ISO Method) (GB/T 17671-2021) [21]. Three replicate specimens were tested for each compressive and flexural strength measurement (n = 3).

Following demolding, all specimens were transferred to a standard curing chamber maintained at a controlled temperature of 20 ± 2 °C and a relative humidity (RH) exceeding 95% for standard curing. Recognizing the time-dependent kinetics governing early-stage hydration reactions in cement-based composites, microstructural development and associated strength evolution accelerate rapidly between 7 days and 14 days. By 28 days, the hydration products converge toward thermodynamic and spatial equilibrium, enabling the matrix to achieve its nominal design strength. Consequently, curing durations of 7 days, 14 days, and 28 days were selected as the temporal benchmarks to map the time-varying evolutionary trajectories of the modified composite systems.

#### 2.3.2. Dynamic Viscoelastic Performance Tests

The dynamic mechanical and viscoelastic properties of the modified cementitious composites were characterized using a high-precision dynamic mechanical analyzer (DMA Q800, TA Instruments, New Castle, DE, USA). Given that the viscoelastic response of multiphase cementitious systems is highly sensitive to external thermal fluctuations and matrix hydration states, the testing environment was strictly maintained at isothermal conditions of 25.0 ± 0.5 °C. Rectangular micro-beam specimens (60 mm × 8 mm × 2.5 mm) (Figure 2c) of a designated age were selected and tested using the three-point bending dynamic loading method (support span = 50 mm). Six independent specimens were tested for each DMA measurement (n = 6). Before DMA testing, all specimens were dried at 60 °C for 24 h in accordance with the instrument testing requirements and were subsequently cooled to the testing temperature under laboratory conditions. This pretreatment was used to substantially reduce evaporable free water and minimize differences in moisture condition among the specimens. However, drying at 60 °C does not remove chemically bound water and may not completely eliminate adsorbed or confined moisture within the fine pore structure.

To ensure stable and repeatable viscoelastic characterization under identical testing conditions, constant dynamic displacement amplitude of 7 μm and frequency sweeps spanning the quasi-static to low-frequency regimes (0.5 Hz to 2.5 Hz) were performed to capture the frequency-dispersive viscoelastic profiles. Temperature sweep tests were conducted using the same DMA three-point bending fixture. The specimens were heated from −50 to 60 °C at a rate of 5 °C/min under an air atmosphere with a displacement amplitude of 7 μm. Each specimen was tested using a single heating cycle without thermal cycling. The dynamic energy-dissipation capacity was quantitatively evaluated using the dimensionless loss factor (tan *δ*), which represents the ratio of viscous energy dissipated via molecular internal friction to the maximum elastic energy stored per cycle, mathematically expressed as:

(1) $$\tan\delta =\frac{E^{\prime \prime }}{E^{\prime }}$$

where *E*′ is the storage modulus (GPa), quantifying the elastic strain energy capacity and representing the dynamic structural stiffness of the composite; *E*″ is the loss modulus (GPa), denoting the viscous dissipation of strain energy into heat via irreversible microstructural shear deformation or polymer chain relaxation. The loss factor tan *δ* serves as an intrinsic viscoelastic descriptor; higher tan *δ* values indicate greater damping capacity, arising from interfacial polymer-cement sliding, viscoelastic molecular relaxation within the polymeric phases, and hysteretic energy consumption at microcrack tips.

#### 2.3.3. Multiscale Microstructural Characterization

To decode the physical and chemical mechanisms governing the macroscopic mechanical and dynamic damping performance, multiscale microstructural characterization was implemented. High-resolution morphological imaging was performed via field-emission scanning electron microscopy (FE-SEM, Quanta FEG 250, FEI Company, Hillsboro, OR, USA). SEM investigations were focused on the interfacial transition zone (ITZ) morphology, matrix densification, and the microscale spatial dispersion of the polymeric damping phases within the hydration product network. SEM specimens were collected from the fractured sections of the 28-day specimens after the mechanical tests. The specimens were sputter-coated with a thin gold layer before observation.

Complementarily, mercury intrusion porosimetry (MIP, AutoPore IV 9510, Micromeritics Instrument Corp., Norcross, GA, USA) was employed to obtain cumulative pore-volume and pore-size distribution curves. Approximately 5 mm cubic specimens were collected from the interior of the mortar specimens after 28 days of curing. Hydration was terminated using absolute ethanol, followed by vacuum drying before testing. These measurements yielded key microstructural descriptors, including cumulative porosity, pore-size distribution, and critical threshold pore diameters. These multiscale characterizations established a rigorous mechanistic foundation for clarifying the “microstructure–porosity–damping” coupling relationships in hybrid-modified cementitious systems.

In addition, our previous Fourier Transform Infrared Spectroscopy (FTIR) study demonstrated the interaction between the functional groups of XSBR latex and cement hydration products, providing indirect evidence for the proposed interfacial physicochemical interactions. The present work therefore adopts these previously verified findings as the theoretical basis for interpreting the observed microstructural evolution.

## 3. Results and Analysis

### 3.1. Mechanical Properties and Microstructural Interpretation

#### 3.1.1. Strength Evolution and Synergistic Weakening Induced by Viscoelastic Modifiers

**Figure 3:**
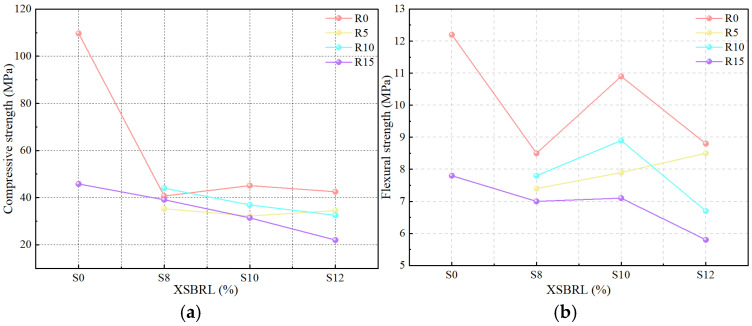
Effect of XSBRL and rubber powder content on the mechanical properties: (**a**) Compressive strength; (**b**) Flexural strength. (Note: The XSBRL content shown on the horizontal axes of all figures is expressed as the dosage of wet latex emulsion.).

Figure 3 illustrates the 28-day compressive and flexural strengths of the cementitious composites modified with XSBRL and CR-10 powder. Both polymeric modifiers reduce the macroscopic load-bearing capacity. Although incorporating XSBRL inevitably reduced compressive strength compared with the unmodified control, the modified mixtures still satisfied the mechanical requirements for many non-load-bearing or vibration-control applications. Crucially, this strength degradation is not a simple linear superposition of two independent weakening mechanisms. Instead, XSBRL and CR-10 interact synergistically within the cementitious skeleton, altering hydration kinetics, pore structure, and stress transfer efficiency across the modified interfaces.

For compressive strength (Figure 3a), the retarding and structural-disruption effects of XSBRL are particularly pronounced. In the absence of CR-10 (the R0 series), incorporating 8 wt.% XSBRL precipitates a sharp 62.7% reduction in compressive strength (from 110.0 MPa for S0R0 to 41.0 MPa for S8R0). Beyond this threshold, increasing the XSBRL dosage to 10% and 12% results in marginal fluctuations (45.0 MPa and 42.5 MPa, respectively), indicating that the structural integrity of the rigid cementitious matrix reaches a stable, albeit severely weakened, equilibrium.

Upon the introduction of CR-10 powder, the mechanical response exhibits a non-monotonic trend. At a fixed XSBRL dosage of 8 wt.%, S8R10 exhibits a 23% increase in compressive strength compared to S8R5. This localized recovery is attributed to the physicochemical coupling between the two modifiers: the film-forming XSBRL encapsulates and anchors the CR-10 particles, where the strong carbon–chlorine (C–Cl) bonds of CR-10 promote local stress redistribution and enhance shear-stress transfer across the polymer–cement interface. However, exceeding this optimal replacement level (the R15 series) results in a sharp 33% loss in strength relative to S8R10. At this stage, the excessive concentration of low-modulus polymeric inclusions disrupts the continuity of the hydration skeleton, causing the flexible inclusions to dominate the failure path.

The flexural strength profiles (Figure 3b) exhibit a more pronounced recovery at moderate dosages. Single doping with 8 wt.% XSBRL reduces the flexural strength by 31% relative to S0R0 (to 8.5 MPa). However, when the dosage is increased to 10 wt.%, the flexural strength recovers to 10.9MPa (a 28.2% recovery relative to S8R0). This recovery indicates that under a tensile-shear stress field, the continuous polymer network established at higher latex concentrations successfully bridges microcracks, compensating for the matrix porosity. In the hybrid-modified systems, this beneficial bridging effect is optimized at S10R10, which exhibits a 12.7% higher flexural strength than S10R5. This synergy suggests that the high viscoelastic shear-resistance of the CR-10 phase and the bridging capacity of the XSBRL film act cooperatively to delay microcrack propagation. Beyond a threshold of 12 wt.% XSBRL (S12 series), however, the excessive accumulation of polymeric phases severely undermines matrix continuity, accelerating flexural failure.

#### 3.1.2. Mechanisms Responsible for Strength Degradation

The deterioration of macroscopic mechanical properties originates from the competitive coupling of three distinct mechanisms: hydration retardation, pore-structure coarsening, and elastic modulus mismatch.

First, XSBRL particles compete with cement hydration products for available space for growth. Anionic functional groups (e.g., carboxylates) in the latex surfactant preferentially adsorb onto unhydrated cement grains, delaying the dissolution of clinker phases and restricting the nucleation of C–S–H gels and Ca(OH)_2_ crystals. This chemical inhibition reduces the volume fraction and spatial continuity of the rigid hydration framework [15]. Concurrently, the surfactants in the latex stabilize entrained air bubbles during mixing, coarsening the pore structure and facilitating strain-energy localization at pore boundaries under external loads.

Second, the elastic modulus of the CR-10 polymer filler is orders of magnitude lower than that of the hardened paste, causing these particles to behave mechanically as “soft inclusions” or flexible voids. Under external loading, the severe modulus mismatch between CR-10 powders and the surrounding paste induces localized stress concentrations around the interfacial transition zone (ITZ), promoting micro-debonding and accelerating crack initiation [22,23]. When the polymer filler content exceeds critical thresholds, these weak ITZ regions interconnect, forming preferential pathways for macro-crack propagation. Consequently, the mechanical degradation is governed by a cooperative mechanism involving chemical hydration inhibition, physical dilution, and mechanical modulus mismatch.

#### 3.1.3. Strength Development Kinetics at Different Curing Ages

**Figure 4:**
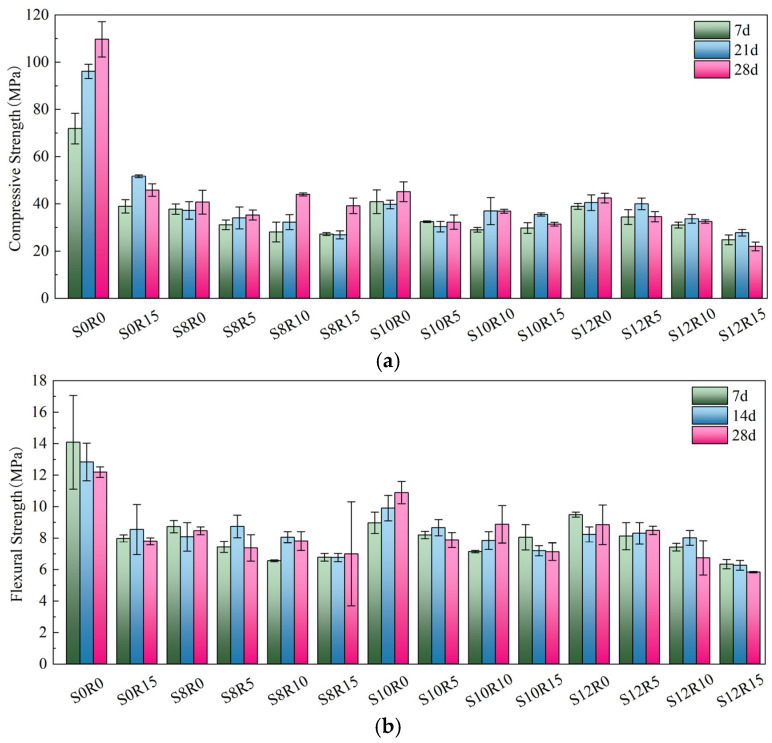
Comparison of Mechanical Strength in Composite-Modified Concrete at Different Ages: (**a**) Compressive strength (7 days, 14 days, 28 days); (**b**) Flexural strength (7 days, 14 days, 28 days). Note: Error bars in all figures represent one standard deviation (SD).

Figure 4 presents the compressive and flexural strength development of the composites at different curing ages (7 days, 14 days, and 28 days). All mixtures exhibit a progressive strength gain over time, confirming that the polymer modification does not arrest the intrinsic hydration-driven densification. However, XSBRL and CR-10 alter the kinetics of strength development through fundamentally different mechanisms.

XSBRL significantly suppresses the rate of early-stage strength development. For the single-latex series (S8R0, S10R0, and S12R0), the 28-day compressive strengths increase by only 7.61%, 10.29%, and 8.97%, respectively, relative to their 7-day values, which is dramatically lower than the 52.8% increase observed in the control S0R0. This kinetic retardation is governed by the chemical adsorption of anionic latex polymer chains onto hydrated ionic sites, which imposes steric hindrance and electrostatic shielding, thereby postponing early-stage matrix densification [15].

Conversely, at a constant XSBRL dosage of 8 wt.%, the mixtures incorporating higher CR-10 contents maintain significant late-age strength growth, with the compressive strength of S8R10 and S8R15 increasing by 56.58% and 43.59%, respectively, from 7 days to 28 days. Because CR-10 is introduced as a solid powder rather than a liquid phase, its chemical retardation is minimal compared to XSBRL. Crucially, the polar C–Cl groups on the CR-10 surface exhibit mild physicochemical affinity with the cement hydration products, allowing late-stage hydration products to nucleate and deposit continuously around the polymer boundaries. This ensures that while the absolute strength of CR-10-doped systems is lower due to physical dilution, their late-age strength growth kinetics remain largely uninhibited, yielding a more stable long-term mechanical skeleton.

### 3.2. Damping Performance Test Results

#### 3.2.1. Effect of XSBRL and CR-10 Powder Content on Loss Factor

**Figure 5:**
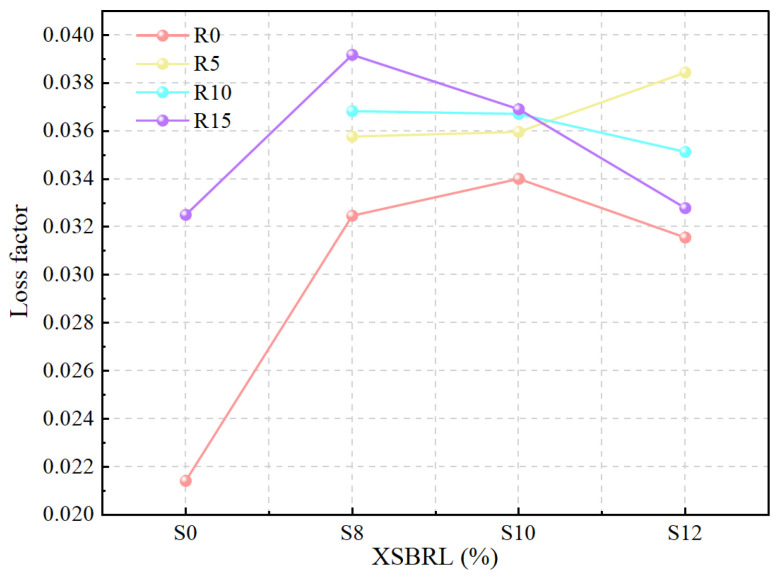
Loss Factor of Specimens at 28 days of Age and 1.5 Hz Frequency.

Figure 5 presents the 28-day loss factor (tan *δ*) of the cementitious composites at a dynamic frequency of 1.5 Hz as a function of XSBRL dosage and CR-10 aggregate replacement. Both viscoelastic modifiers markedly enhance the matrix’s energy-dissipation capacity, exhibiting a highly non-linear, composition-dependent synergistic interaction.

In the modifier-free group (R0), the pristine reference (S0R0) exhibits a baseline tan *δ* of only 0.021, characteristic of a highly rigid elastic response. Incorporating 8 wt.% (S8R0) and 10 wt.% (S10R0) XSBRL elevates the loss factor to 0.032 (+52%) and 0.034 (+62%), respectively. This damping gain is governed by the coalescence of XSBRL particles into a continuous three-dimensional viscoelastic macromolecular network across the boundaries of hydration products, thereby generating pronounced interchain friction and shear hysteresis under dynamic strain [24]. At 12 wt.% XSBRL (S12R0), however, the loss factor marginally declines to 0.0315, indicating that excessive polymer accumulation over-encapsulates the hydration sites, suppressing interfacial micro-shear slippage.

The incorporation of CR-10 powder further elevates the dynamic energy dissipation. Even without XSBRL, the single-doped S0R15 achieves a tan *δ* of 0.0325 (+55% vs. S0R0) due to the intrinsic viscoelastic shear relaxation and elastic hysteresis of the polymer filler. Under dual-modification, the damping performance is substantially amplified; S8R15 reaches a global maximum of 0.0392, representing a 21% synergistic gain over the single-doped S0R15. This enhancement is driven by a polar molecular coupling mechanism: the polar carboxyl groups (–COOH/–COO–) of XSBRL and the highly polar carbon–chlorine (C–Cl) bonds of CR-10 establish strong dipole–dipole attractions and hydrogen bonding at the interface. This interfacial optimization maximizes dynamic shear-stress transfer from the cement paste to the CR-10 core, fully activating its viscoelastic energy-dissipation capacity [18,25]. Within the 10 wt.% XSBRL window, the R5–R15 series establishes a robust damping plateau (tan *δ*, 0.036–0.037). However, at 12 wt.% XSBRL, the loss factors of the R10 and R15 groups decline by 5% and 13%, respectively. This regression represents “interfacial anchoring passivation,” in which an excess of polymer film fills the ITZ nanopores, thereby restricting the physical slip space essential for micro-shear damping.

#### 3.2.2. Frequency Dependence of the Loss Factor

**Figure 6:**
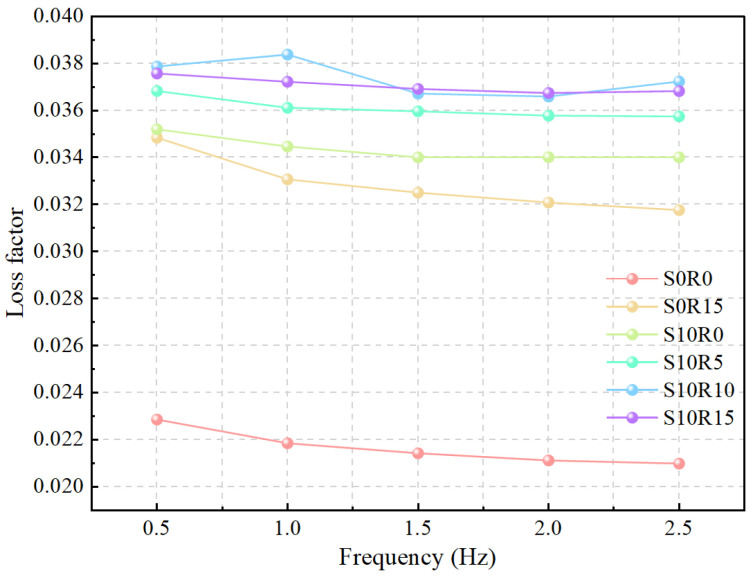
Change in Specimen Loss Factor with Frequency.

Figure 6 depicts the evolution of tan *δ* over the frequency range of 0.5 to 2.5 Hz. All modified systems exhibit a monotonic decrease in the loss factor with increasing frequency, consistent with classical linear viscoelastic relaxation theory. At lower frequencies, polymer chains and segments possess sufficient relaxation time to undergo coordinated conformational rearrangements and interchain slippage, maximizing the viscous dissipation component. As the dynamic excitation frequency accelerates, the alternating shear strain rate outpaces the conformational rearrangement rate of the polymer segments, suppressing interfacial slippage and progressively stiffening the dynamic response.

Importantly, the unmodified S0R0 reference drops by 8% across the frequency spectrum (from 0.0228 at 0.5 Hz to 0.0210 at 2.5 Hz). In contrast, the synergistically modified composites not only maintain significantly higher absolute damping levels but also exhibit remarkably minimized frequency sensitivity. Notably, the S10R10 group varies marginally, from 0.0378 (0.5 Hz) to 0.0372 (2.5 Hz), with a total fluctuation of less than 4%. This damping stability within 0.5–2.5 Hz demonstrates a highly reliable intrinsic energy-dissipation capability at the material scale. While this low-frequency regime reflects key baseline dynamic properties relevant to primary structural vibration modes, full-scale structural damping performance ultimately depends on systemic boundary conditions as well as broader frequency and strain amplitude variations, which warrant further multiscale validation.

#### 3.2.3. Temperature Sensitivity of the Loss Factor

**Figure 7:**
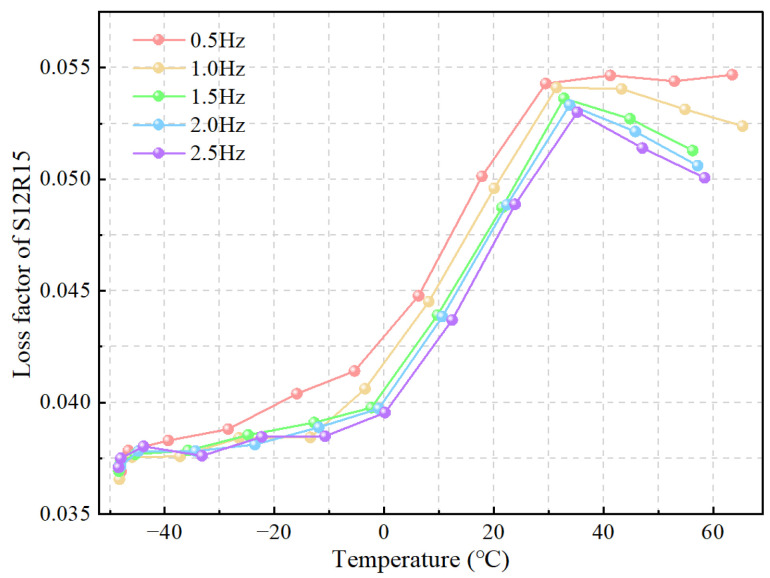
Change in Specimen Loss Factor with Temperature.

Figure 7 displays the temperature-dependent loss factor of S12R15 over the temperature range −50 °C to 60 °C. The curves trace an inverted-U profile, directly reflecting the glass transition (T_g_) relaxation dynamics of the polymeric phases.

In the low-temperature regime (−50 °C to −20 °C), the loss factor remained locked in a stable, low-energy state (tan *δ*, 0.037–0.039). Within this range, the thermal kinetic energy is insufficient to overcome the potential barriers of the macromolecular segments; the polymer chains remain frozen in the glassy state, meaning residual damping is dominated by the elastic shear of the rigid cement crystal framework.

Above 0 °C, the loss factor increased steeply, reaching a high-efficiency damping plateau of 0.053–0.055 centered around 30 °C, yielding a 40–45% gain over the low-temperature baseline. This optimal temperature window coincides with the overlapping glass transition zones of XSBRL (T_g_, −20 °C to 20 °C) and chlorinated rubber (T_g_, 10 °C to 30 °C), wherein the main-chain and side-chain segments of both polymers acquire sufficient activation energy for cooperative, large-scale viscoelastic relaxation [6,19]. The resulting viscous resistance and intermolecular friction convert dynamic kinetic energy into heat. Beyond 40 °C, the polymer transitions into the rubbery state; segmental mobility becomes unrestricted, reducing internal viscosity and dynamic stiffness, which leads to a decline in tan *δ*. Crucially, the optimal operating temperature range of 20–40 °C aligns with standard environmental engineering exposures, providing a solid physical foundation for real-world vibration control.

#### 3.2.4. Age-Dependent Evolution of the Loss Factor

**Figure 8:**
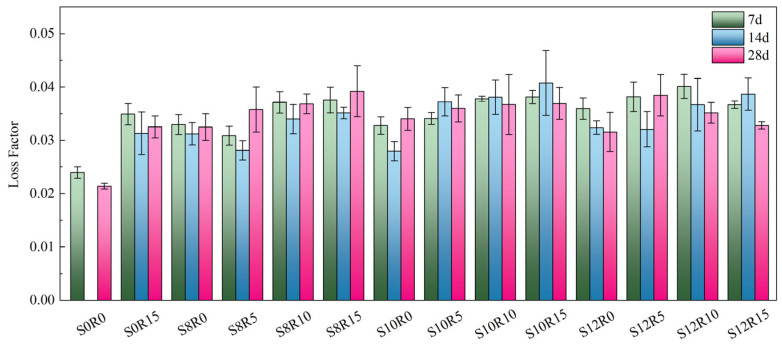
Change in Specimen Loss Factor at Different Ages. Note: Error bars in all figures represent one standard deviation (SD).

Figure 8 presents the age-dependent evolution of the loss factor at 7, 14, and 28 days for all specimen groups. Hydration age exerts a complex, competitive regulatory effect on the dynamic viscoelastic networks.

For the control S0R0, the loss factor declined continuously from 0.024 (7 days) to 0.021 (28 days) due to hydration-driven matrix densification: as C–S–H gels fill the capillary pores, the ascending framework rigidity suppresses intergranular friction and interfacial slip. Conversely, the modified systems maintain high damping levels throughout the curing history. For instance, the S8R15 group stabilizes at 0.037 (7 days), 0.035 (14 days), and 0.039 (28 days), which are 54% and 86% higher than the corresponding S0R0 benchmarks, demonstrating that the viscoelastic phase successfully counteracts hydration-induced damping decay.

Most co-modified mixtures exhibit a non-monotonic age evolution: a slight decline between 7 days and 14 days, followed by a secondary rebound at 28 days (e.g., S8R5 drops from 0.031 to 0.028, then rebounds to 0.036). This trend reflects the kinetic competition between early-stage hydration and late-stage polymer film formation. From 7 days to 14 days, the rapid precipitation of crystalline hydration products around the dispersed polymer phases restricts interfacial sliding, compressing the viscoelastic shear space. In the later stage (14–28 days), as free water is progressively consumed, the XSBRL undergoes irreversible coalescence, forming a continuous 3D viscoelastic film that spans crystal boundaries and ITZ micropores [24]. Concurrently, the polar groups on the CR-10 surfaces facilitate the orderly deposition of late-stage hydration products, establishing a cross-scale dynamic viscoelastic network that overcomes matrix stiffening and drives the 28-day damping rebound.

## 4. Microscopic Test Results and Mechanism Analysis

### 4.1. Mercury Intrusion Porosimetry (MIP) Results and Energy-Dissipation Mechanisms

Pore structure dictates both the mechanical integrity and damping capacity of cementitious composites. Porosity, pore-size distribution, and critical pore size collectively govern internal stress propagation, interfacial slip, and viscoelastic shear hysteresis. While high overall porosity generally degrades static mechanical strength, a well-developed, multiscale pore network—especially capillary pores in the 10–100 nm range—maximizes the internal specific surface area, providing an efficient medium for microscale interfacial friction and viscoelastic energy dissipation [18,19,25,26].

Following cement chemistry conventions, pores within the hardened matrix are classified as gel pores (<10 nm), small capillaries (10–100 nm), large capillaries (100–1000 nm), and macropores (>1000 nm). A high density of gel pores and small capillaries, reflecting a well-developed calcium silicate hydrate (C–S–H) gel structure, provides abundant molecular-contact surfaces that facilitate interfacial sliding friction. Conversely, mitigating macropores and macroscopic defects suppresses localized stress concentration, preventing unstable microcrack propagation under dynamic cyclic loading [25,26].

#### 4.1.1. Pore Refinement and Topological Transition Under Composite Modification

**Figure 9:**
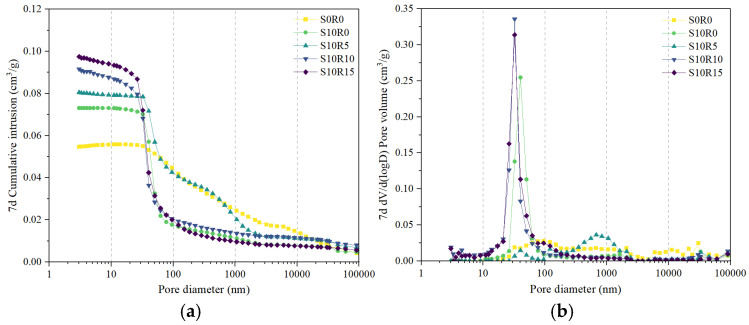
Pore structure of S10-series composites at 7 days characterized by MIP: (**a**) cumulative mercury intrusion volume; (**b**) differential pore-size distribution.

**Figure 10:**
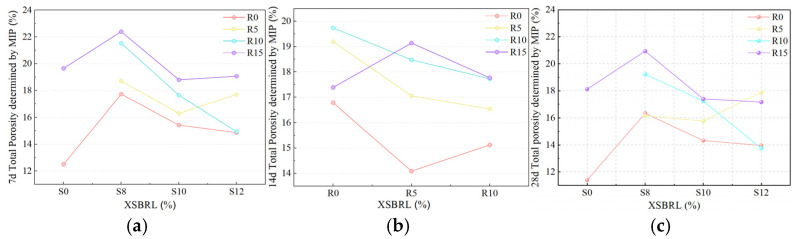
Total porosity of modified composites determined by MIP across different curing ages: (**a**) 7 days; (**b**) 14 days; (**c**) 28 days.

**Figure 11:**
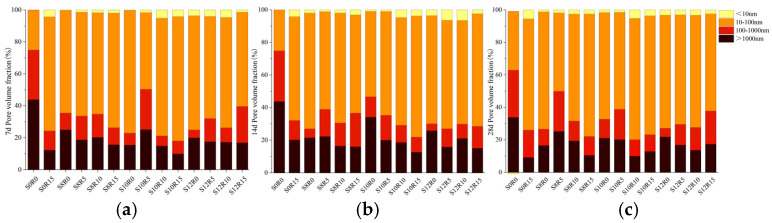
Pore volume fractions of different pore-size ranges at different curing ages: (**a**) 7 days; (**b**) 14 days; (**c**) 28 days.

Figure 9, Figure 10 and Figure 11 present the cumulative porosity, pore-size differential distributions, and pore volume fractions for the modified composites. Compared with the control specimen (S0R0), the binary-modified systems containing XSBRL and CR-10 powder exhibited concurrent increases in total porosity and significant pore-size refinement. The critical pore size of the modified specimens concentrated in the 30–40 nm range, representing a distinct shift toward smaller diameters compared to S0R0.

This shift indicates a topological transition from macropores to fine capillary pores during matrix hardening. Notably, the volume fraction of small capillaries—the primary locus of high-efficiency damping—increased substantially (e.g., S10R15 exhibited a 2.04-fold increase in small-capillary volume relative to S0R0), while harmful macropores (>1000 nm) were suppressed. This refinement confirms that the synergistic action of XSBRL and CR-10 powder effectively reshapes and partitions macroscopic defects within the cement matrix.

#### 4.1.2. Individual Effects of Viscoelastic Modifying Components

Regarding the individual effect of XSBRL, specimens S8R0, S10R0, and S12R0 exhibited increases in porosity of 41.79%, 23.42%, and 18.96%, respectively, compared to S0R0, whereas their macropore volumes decreased by 44.11%, 65.60%, and 55.22%, respectively. This pore-refining mechanism is twofold: first, surfactants in the latex entrain highly dispersed, fine microvoids during mixing; second, during cement hydration and water consumption, the latex undergoes phase separation, coalescing into viscoelastic polymeric films on the surface of hydration products and within the ITZ [24]. These polymer-rich films physically fill and partition large voids, converting them into fine capillary pores. However, at 12 wt.% XSBRL, this trend reversed; local agglomeration of excessive latex particles can coat unhydrated cement grains, retarding hydration and leaving water-filled microvoids that collapse into coarser pores upon drying. This threshold effect explains the corresponding decline in macroscopic damping at excessive latex contents.

For the single-doped chlorinated rubber specimen (S0R15), total porosity increased by 56.80%, the small capillary volume increased by 1.79-fold, and the macropore volume decreased by 72.45% relative to S0R0. Although the inclusion of CR-10 powder introduces stable micro-interfaces through physical incorporation of the powder and local air entrainment, its polar C−Cl bonds ensure better thermodynamic compatibility with the hydrated phase than that of traditional non-polar rubber powder. Under dynamic stress waves, these highly compliant, elastic CR-10 inclusions induce localized stress redistribution and shear-strain concentration at the matrix-particle interfaces, thereby enhancing energy dissipation through elastic deformation and promoting interfacial friction along the tortuous micropore boundaries [27,28].

#### 4.1.3. Interfacial Coupling: Synergy Versus Passivation

When XSBRL and CR-10 powder are combined, their influence on the pore structure exhibits distinct non-linear regimes governed by interfacial coupling interactions:

(1) Synergistic Damping Region (0–5% CR-10): Within this range, both total porosity and small capillary volume increase stepwise. The polar C−Cl bonds of the CR-10 powder interact with the carboxyl groups of XSBRL via dipole–dipole interactions and hydrogen bonding. This chemical compatibility allows the coalesced polymer film and CR-10 particles to interweave within the ITZ, establishing a highly integrated, multiscale viscoelastic network. This configuration optimizes dynamic shear transfer and interfacial slip, facilitating maximum viscoelastic energy dissipation.
(2) Interfacial Anchoring Passivation Region (10–15% CR-10): At higher CR-10 concentrations, although the capillary volume remains high, the rate of porosity growth plateaus. The coexistence of high-content XSBRL and CR-10 leads to an excessively thick polymer–rubber interphase that overfills the micro- and nano-pores within the ITZ. This “over-cementation” excessively strengthens the static interfacial bond, restricting microscopic relative slip. Without sufficient clearance for interfacial shear deformation, the friction-based energy-dissipation pathway is passivated, resulting in the non-linear decline in the dynamic loss factor observed at high composite dosages.

#### 4.1.4. Aging-Controlled Hydration and Film-Formation Competition

**Figure 12:**
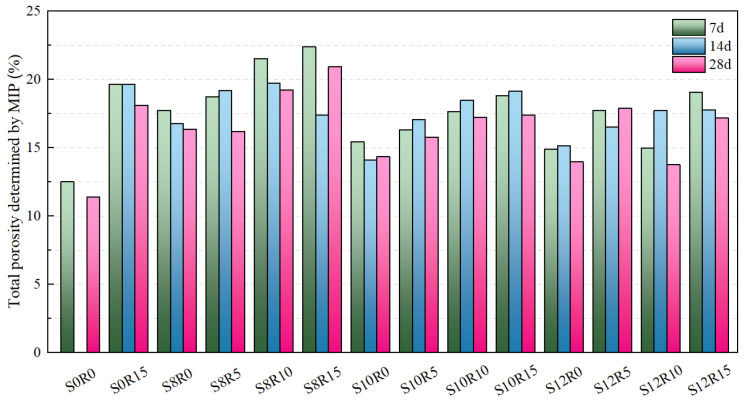
Evolution of total porosity of modified composites as a function of hydration age (7 days, 14 days, and 28 days).

Figure 12 illustrates the evolution of composite porosity as a function of hydration age. During early hydration (7–14 days), rapid crystal growth subdivides large pores into capillary networks, while evaporation of free water causes minor fluctuations in porosity. During late hydration (14–28 days), the continuous accumulation of hydration products fills capillary spaces, stabilizing the porosity.

This microstructural evolution correlates directly with the macroscopic loss factor, which initially decreases and subsequently rebounds. The long-term damping performance is governed by a dynamic competition between the rigid constraints of cement hydration and the flexible viscoelasticity of the coalesced polymer network. Although the joint incorporation of XSBRL and CR-10 powder increases the baseline porosity across all ages (e.g., at 28 days, the porosities of S8R15 and S12R15 remained high at 21.0% and 17.2%, respectively), this permanent, highly capillary network is highly beneficial. It preserves the microstructural framework required for long-term interfacial slip and viscoelastic energy dissipation, successfully preventing the typical damping decay associated with hydration-induced matrix stiffening.

In summary, MIP analysis elucidates the microstructural origins of the composite’s superior damping capacity: namely, the topological refinement of the pore network, the formation of a stable capillary matrix, and the synergistic regulation of interfacial compliance and sliding freedom within the ITZ. Concurrently, the interfacial anchoring passivation mechanism explains the non-linear attenuation of energy dissipation when modifier dosages exceed their optimal thresholds.

### 4.2. SEM Microstructural Characterization and Mechanism Analysis

SEM observations were performed on the fracture cross-sections of four representative specimens—S0R0, S0R15, S12R0, and S12R15—to illustrate the influence of each component and their synergistic effect on the interfacial microstructure, including the phase distribution, interfacial adhesion state, and microstructural energy-dissipation pathways within the modified composites. These specimens represent the reference material, rubber powder modification, XSBRL modification, and their combined modification, respectively.

#### 4.2.1. Reference Matrix (S0R0): Brittle Interfacial Response

**Figure 13:**
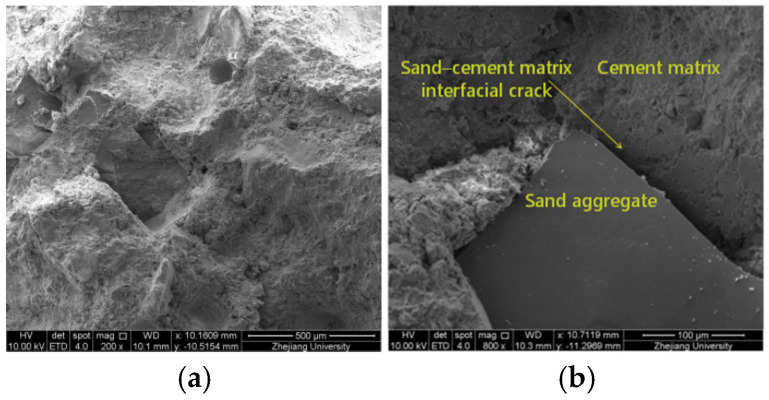
Microstructural morphology of the control specimen (S0R0): (**a**) dense hydration skeleton within the bulk cement matrix; (**b**) smooth, continuous microcracks along the sand-cement ITZ.

The microstructural morphology of the control specimen (S0R0) is shown in Figure 13. The cement matrix displays a highly dense, continuous hydration skeleton composed of crystalline hydration products (Figure 13a), reflecting typical elastic–rigid mechanical features. However, prominent, smooth, and continuous microcracks are observed along the aggregate-cement paste boundary (Figure 13b). This flat interfacial transition zone (ITZ) morphology indicates weak physical adhesion and pronounced interfacial brittleness. Under dynamic loading, stress waves propagate rapidly through this rigid hydration skeleton and along the unanchored boundary defects without triggering substantial micro-mechanical slip or shear friction. Consequently, internal interfacial energy dissipation remains negligible, resulting in a low baseline macroscopic loss factor (≈0.021).

#### 4.2.2. Rubber-Modified Matrix (S0R15): Deformable Inclusions and Interfacial Friction

**Figure 14:**
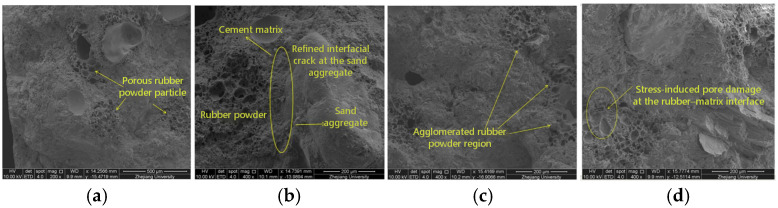
Microstructural morphology of the CR-modified specimen (S0R15): (**a**) porous CR-10 particle embedded in the cement matrix; (**b**) refined interfacial cracks near sand aggregate boundaries; (**c**) localized agglomeration of CR-10 powders; (**d**) stress-induced pore damage at the rubber-matrix interface.

The incorporation of CR-10 powder fundamentally alters the matrix morphology (Figure 14). Unlike conventional non-polar rubber powder, the polar chlorinated rubber (CR-10) particles disperse as discrete, microporous elastic inclusions within the matrix, generating localized microvoids that partition the matrix and shift the pore-size distribution toward finer capillary ranges (Figure 14a), consistent with the MIP results. When located adjacent to sand aggregates, the CR-10 particles interrupt the continuous growth of massive portlandite crystals, reducing the continuity of interfacial through-cracks (Figure 14b). Under dynamic excitation, the low elastic modulus and high compliance of the CR-10 phase induce localized stress redistribution and shear strain concentration at the matrix-particle boundaries, converting mechanical vibration into thermal energy via viscoelastic shear deformation and micro-slip friction.

However, excessive CR-10 content leads to significant particle agglomeration (Figure 14c), creating localized soft zones and severe stress concentration. Stress-induced micro-pore damage is observed at the rubber-matrix interfaces (Figure 14d), which compromises the static load-bearing capacity and confirms that the damping contribution of CR-10 is governed by a threshold.

#### 4.2.3. XSBRL-Modified Matrix (S12R0): Polymer Bridging and Viscous Shear

**Figure 15:**
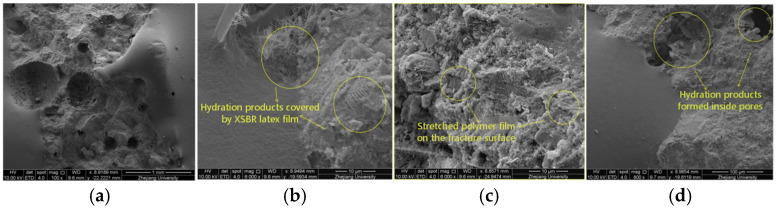
Microstructural morphology of the latex-modified specimen (S12R0): (**a**) spherical polymeric microvoids; (**b**) hydration products encapsulated by the coalesced XSBRL film; (**c**) elongated polymer fibrils bridging a microcrack on the fracture surface; (**d**) secondary hydration products growing within a polymer-lined void.

Figure 15 displays the microstructural features of the XSBRL-modified system (S12R0). The surfactant-induced air-entrainment effect associated with the 12 wt.% latex dosage introduces isolated, spherical micro-voids (Figure 15a) without generating continuous interfacial cracks. At the microscale, the coalesced XSBRL forms a continuous viscoelastic membrane that encapsulates the hydration products (C–S–H and ettringite) (Figure 15b).

Upon tensile fracturing, this polymer film is drawn into elongated fibrils spanning the microcracks (Figure 15c). This morphology provides direct microstructural evidence of a robust polymer crack-bridging mechanism [29], which restrains microcrack nucleation and dissipates substantial strain energy via macromolecular chain stretching and viscous sliding. Concurrently, newly formed hydration products are observed inside the polymeric microvoids (Figure 15d), suggesting that the latex film maintains a local humid environment conducive to continuous cement hydration. Although a 12 wt.% latex dosage is insufficient to form a fully continuous 3D polymer network throughout the bulk paste, localized encapsulation and flexible bridging provide high-efficiency viscous shear pathways, increasing the loss factor by 49.6% relative to the control.

#### 4.2.4. Composite-Modified Matrix (S12R15): Interfacial Chemical Anchoring

**Figure 16:**
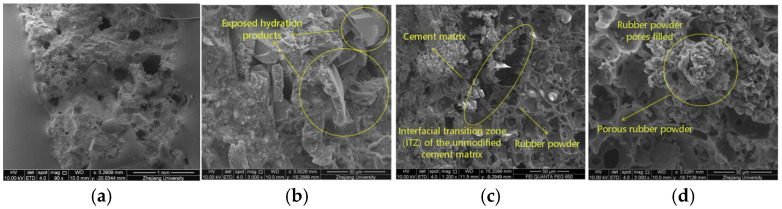
Microstructural morphology of the co-modified specimen (S12R15): (**a**) synergistically modified dense microstructure; (**b**) C–S–H gels and polymer films interweaving at the interface; (**c**) highly densified ITZ showing a “bristly” hybrid interfacial layer; (**d**) internal pores of a CR-10 particle filled with secondary hydration products.

Co-modification with XSBRL and CR-10 powder produces a highly dense, synergistically reinforced microstructure (Figure 16). Unlike the distinct, weakly bonded boundaries in S0R15, the interfaces between the CR-10 particles and the cement matrix in S12R15 are highly diffuse and seamlessly integrated. Abundant hydration products deposit directly onto both the CR-10 surfaces and within its internal pores (Figure 16b,d), forming a highly interlocking, dense “bristly” interfacial layer that significantly densifies the ITZ (Figure 16c).

This microstructural refinement originates from a synergistic chemical-physical anchoring mechanism: the polar C−Cl bonds on the CR-10 surface exhibit high thermodynamic and polar compatibility with the hydrophilic latex and cement hydration products. Concurrently, the active carboxyl groups (–COOH) of the XSBRL chains coordinate with Ca^2+^ ions in the pore solution, forming stable calcium carboxylate ion bridges (–COO–Ca–OOC–) [15,30]. This chemical coupling anchors the interpenetrated polymer–rubber phase directly onto the C–S–H gels, transitioning the interfacial mechanics from simple, weak mechanical interlocking to a robust, chemically bonded hybrid network. This structural refinement optimizes dynamic shear-stress transfer, preventing premature interfacial debonding while maximizing microscale friction and viscoelastic energy dissipation, thereby leading to the optimal damping performance observed in the co-modified composites.

#### 4.2.5. Multiscale Energy-Dissipation

The SEM observations, synthesized with the MIP and macroscopic dynamic data, reveal a hierarchical, three-tier energy-dissipation framework governing the high-damping behavior of the co-modified cementitious composites:

(1) Pore-scale friction: The co-incorporation of XSBRL and CR-10 powder partitions macroscopic voids into a stable, high-specific-surface-area capillary network (10–100 nm). This structural transition establishes an expansive microstructural substrate for capillary-pore wall friction and micro-slip sliding under dynamic loads.
(2) Film-scale viscoelastic dissipation: The coalesced XSBRL viscoelastic films within the bulk paste and along the ITZ act as flexible dampers. These films bridge microcracks and dissipate dynamic strain energy through macromolecular chain relaxation and viscous shear, with the elongated fibrils serving as clear structural evidence.
(3) Chemical-bond-scale anchoring: Polar–polar compatibility (C−Cl and polar matrix) and ionic coordination (–COO–Ca–OOC–) convert weak physical boundaries into a chemically integrated hybrid interphase. This stable anchoring enables high-efficiency shear-stress transfer without interfacial peeling, maximizing viscoelastic hysteretic dissipation and preventing typical hydration-induced damping decay.

These three mechanisms operate across complementary length scales and act cooperatively to constitute the microscopic physical basis of the multiscale energy-dissipation system. The resulting chain of microstructural evidence provides a complete mechanistic explanation for the significant enhancement in macroscopic damping performance of the modified cementitious composite.

## 5. Conclusions

This study systematically investigated the age-dependent mechanical performance, damping capacity, and microstructural evolution of cementitious composites modified with XSBRL and CR-10 powder. By integrating DMA, MIP, and SEM, the synergistic energy-dissipation mechanisms of the polymer–rubber hybrid phase and their evolution with hydration age were elucidated. The primary conclusions are as follows:

(1) While both modifiers degrade static mechanical strength due to cement-hydration retardation and matrix dilution, a critical dosage-dependent optimization window exists. At 10 wt.% XSBRL (S10) and 10 wt.% CR-10 (R10), the composite achieves a highly balanced mechanical state. XSBRL controls macroscopic defects via latex-film partitioning, whereas the compliant, polar CR-10 particles homogenize internal stress distribution. Notably, although single-doped XSBRL flatlines the late-age strength-gain curve (growing only 10.3% from 7 days to 28 days) due to surfactant-induced retardation, the inclusion of CR-10 in co-modified systems (e.g., S8R10) preserves a high late-age strength-growth potential (up to 56.6%), effectively reconciling the typical trade-off between mechanical stiffness and damping capacity.
(2) The loss factor (tan *δ*) is synergistically enhanced by the binary polymer–rubber system, with S8R15 achieving a peak loss factor of 0.0392 (an 86.7% increase over the control). This synergy arises from the interweaving of the coalesced XSBRL film and compliant CR-10 elastic inclusions within the ITZ, stabilized by polar–polar (C−Cl and carboxyl groups) compatibility and ionic coordination. The dynamic damping of the co-modified system exhibits stable broadband characteristics (fluctuating <4% across 0.5–2.5 Hz), a temperature-dependent viscoelastic transition peaking within 20–40 °C, and a non-monotonic age-dependent evolution (dipping at 7–14 days before recovering by 28 days) governed by the competition between rigid cement-hydration constraints and flexible polymer-film coalescence.
(3) Microstructural and pore network characterization confirms a pronounced topological transition. Both modifiers cooperatively refine the pore structure, partitioning harmful macropores (>1000 nm) into small capillaries (10–100 nm), thereby expanding the specific surface area for microscale slip. SEM observations corroborate that XSBRL shifts the CR-10 –cement interface from weak physical interlocking to a dense, chemically anchored “bristly” hybrid layer. This chemical anchoring optimizes shear stress transfer, facilitating a three-tier energy-dissipation system: pore-scale friction along refined capillary walls, film-scale viscous shear via elongated polymer fibrils bridging microcracks, and chemical-bond-scale anchoring that prevents interface peeling.
(4) Comprehensively balancing strength retention, damping capacity, and pore refinement, the S10R10 formulation cured for 28 days represents the recommended balanced mix design for structural-functional concrete components, offering a practical benchmark for high-damping cementitious composites.
(5) It should be emphasized that the present findings characterize the baseline viscoelastic dissipation behavior at the material scale within 0.5–2.5 Hz; direct application to system-level structural damping under wide-spectrum dynamic loads (e.g., traffic vibrations or seismic waves) requires further study incorporating broader frequency and strain amplitude variations.

Future efforts will first extend the dynamic strain amplitude range to systematically map the linear viscoelastic region (LVR) boundaries and non-linear damping behaviors under larger excitations. Concurrently, advanced techniques such as digital image correlation (DIC), nanoindentation, and in situ SEM loading will be employed to quantitatively map local strain fields, interfacial stiffness gradients, and crack-tip deflection paths. These multiscale insights will support the formulation of analytical models for predicting age-dependent damping and facilitate the data-driven design of high-damping cementitious mixtures.

## Figures and Tables

**Table 1:** Technical specifications of XSBRL.

Solid Content (%)	Viscosity (mPa.s)	Density (g/cm^3^)	Average Particle Size (nm)	Glass Transition Temperature (°C)	Surface Tension (mN/m)	pH
50–52	35–150	1.01	150	−20–20 adjustable	30–48	7.0–9.0

**Table 2:** Technical specifications for chlorinated rubber powder (CR-10).

Viscosity (mPa.s)	Chlorine Content (%)	Water (%)	Thermal Decomposition Temperature (°C)	Solubility (20% Toluene Solution)	Apparent Density (g/cm^3^)	Particle Size (µm)	Appearance
45–54	65–68	≤0.2	≥105	30–48	1.43–1.50	45–75	White powder

**Table 3:** Mixture proportions of modified cementitious composites (kg/m^3^).

Index	Cement	Effective Water	Added Mixing Water	Fine sand	CR-10	XSBRL	PCE	Defoamer
S0R0	854	256.2	256.20	1708.0	0.0	0.00	0.00	0.000
S0R15	854	256.2	256.20	1451.8	256.2	0.00	11.25	0.000
S8R0	854	256.2	222.04	1708.0	0.0	68.32	11.25	0.150
S8R5	854	256.2	222.04	1622.6	85.4	68.32	11.25	0.150
S8R10	854	256.2	222.04	1537.2	170.8	68.32	11.25	0.165
S8R15	854	256.2	222.04	1451.8	256.2	68.32	11.25	0.165
S10R0	854	256.2	213.50	1708.0	0.0	85.40	11.25	0.165
S10R5	854	256.2	213.50	1622.6	85.4	85.40	11.25	0.165
S10R10	854	256.2	213.50	1537.2	170.8	85.40	11.25	0.180
S10R15	854	256.2	213.50	1451.8	256.2	85.40	11.25	0.180
S12R0	854	256.2	204.96	1708.0	0.0	102.48	11.25	0.180
S12R5	854	256.2	204.96	1622.6	85.4	102.48	11.25	0.180
S12R10	854	256.2	204.96	1537.2	170.8	102.48	11.25	0.195
S12R15	854	256.2	204.96	1451.8	256.2	102.48	11.25	0.195

Note: (1) Added Mixing Water = Effective Water (256.2 kg/m^3^, w/c = 0.30) − 0.5 × wet XSBRL, compensating for the 50% water content in the latex. (2) CR = chlorinated rubber, XSBRL = carboxylated styrene-butadiene rubber latex, PCE = polycarboxylate ether-based superplasticizer.

## Data Availability

The original contributions presented in this study are included in the article. Further inquiries can be directed to the corresponding author.
